# A Review of Current Evidence for the Use of Steroids in the Medical Intensive Care Unit

**DOI:** 10.3390/diagnostics14141565

**Published:** 2024-07-19

**Authors:** Patrick Jenkins, Cory Cross, Tony Abdo, Houssein Youness, Jean Keddissi

**Affiliations:** Section of Pulmonary, Critical Care and Sleep Medicine, The Oklahoma City VA Healthcare System and The University of Oklahoma Health Sciences Center, Oklahoma City, OK 73104, USA

**Keywords:** acute respiratory distress syndrome, COVID-19, septic shock, severe pneumonia, hypercapnic respiratory failure, chronic obstructive pulmonary disease

## Abstract

Systemic steroids are frequently used in critically ill patients for their anti-inflammatory properties. Potential benefits of these agents should be balanced against their known side effects. In this paper, we review trials assessing the use of systemic steroids in common conditions requiring admission to the intensive care unit. These include septic shock, the acute respiratory distress syndrome, severe pneumonia, COVID-19, and hypercapnic respiratory failure due to chronic obstructive pulmonary disease. We will mainly focus on well-conducted randomized controlled trials to determine whether steroids should be administered to critically ill patients presenting with these conditions.

## 1. Introduction

Glucocorticoids (steroids) are potent anti-inflammatory medications that are frequently used in the treatment of critically ill patients. Systemic glucocorticoids, prednisone, methylprednisolone, and hydrocortisone mimic the action of naturally occurring glucocorticoids by binding intracellular glucocorticoid receptors in the cytoplasm. This glucocorticoid-receptor complex regulates transcription of glucocorticoid response elements such as nuclear factor receptor-κβ, which modulates the transcription of proinflammatory cytokines. Glucocorticoids also promote the activity of anti-inflammatory cytokines and inhibit fibroblast production. In addition to their effect on the immune system, glucocorticoids are key mediators of the stress response. They increase cardiac output, increase the responsiveness of vascular adrenergic receptors, and decrease vascular permeability. Glucocorticoids also have significant effects on metabolism, resulting in increased gluconeogenesis and altered metabolism of fat, bone, and protein [1]. In light of these mechanisms, a trial of glucocorticoids is often used in critical illness when dysregulation of the inflammatory response is proposed to underly the pathology in order to limit organ damage, and promote recovery of organ function, with the goal of improving mortality.

However, glucocorticoids are not without serious potential side effects. In the critically ill population, these side effects include but are not limited to new infections, hyperglycemia, and critical illness myopathy. In order to maximize benefit and limit harm, practitioners must be aware of the evidence justifying the use of glucocorticoids.

In this review, we aim to present the evidence justifying the use of glucocorticoids in septic shock, acute respiratory distress syndrome (ARDS), severe pneumonia, severe SARS-CoV-2 infection (COVID), and hypercapnic respiratory failure due to exacerbation of chronic obstructive lung disease (COPD). We will focus on prospective randomized trials as they provide the highest level of confidence for any recommendation.

## 2. Steroids in Septic Shock

Sepsis and septic shock develop when inflammation spreads beyond the initial site of an infection, resulting in a systemic proinflammatory cascade, which overwhelms the body’s anti-inflammatory processes and leads to diffuse cellular injury and multiorgan damage. This inflammatory mechanism provides a rationale for the use of steroids in the treatment of septic shock. Table 1 summarizes the randomized trials addressing this potential over the last several decades.

Descriptions of glucocorticoids in the treatment of sepsis date back to the 1950s [2], with small randomized controlled trials published in the 1960s and 1970s with contradictory results [3]. In a 1988 study, Luce assessed the effect of high-dose methylprednisolone on mortality and the development of ARDS [4]. Seventy-five patients with septic shock were enrolled within 1 to 2 h of admission or development of shock; patients were required to have a positive culture to ultimately be included in the study. Methylprednisolone, 30 mg/kg per dose for a total of 4 doses, was compared to placebo, and no difference in mortality or risk of ARDS was seen.

A decade later, the concept of relative adrenal insufficiency in septic shock patients and systemic inflammation-induced glucocorticoid receptor resistance prompted renewed interest in longer courses of low-dose corticosteroids. In 2002, in a trial published by Annane et al., 300 patients were randomized to low-dose hydrocortisone (50 mg IV every 6 h) plus fludrocortisone (50 mcg daily) compared to placebo, for a total of 7 days [5]. Patients were enrolled early, within 8 h of meeting criteria for septic shock. An ACTH stimulation test was performed in all patients at the time of enrollment. Responders were defined as patients with an increase in the plasma cortisol level of at least 9 mcg/dL. Overall, a significant improvement in the 28-day survival was seen in the steroid group (hazard ratio 0.71, 95% CI: 0.53 to 0.97, *p* = 0.03). No difference in the rate of adverse events was noted. When analyzing the survival based on the response to the ACTH stimulation test, only patients with relative adrenal insufficiency (i.e., non-responders) showed an improvement in survival or hemodynamics with steroids (hazard ratio 0.67, 95% CI: 0.47 to 0.95, *p* = 0.02). No difference was seen in patients without relative adrenal insufficiency.

In 2008, Sprung assessed the use of hydrocortisone (50 mg every 6 h) in septic shock [6]. Fludrocortisone was not used based on the assumptions that hydrocortisone possesses enough mineralocorticoid activity to make the use of fludrocortisone unnecessary, and absorption of oral fludrocortisone in a shock state is variable. A total of 499 patients were randomized, and all had a corticotropin test performed at baseline. Patients could be enrolled up to 72 h after the onset of shock. No difference in survival was seen regardless of the response to the ACTH stimulation test. There was also no difference in the proportion of patients with shock reversal, but faster time to reversal of shock was seen in patients who received steroids. Patients treated with hydrocortisone had increased incidence of new infections, hyperglycemia, and hyponatremia.

In 2018, two larger placebo-controlled studies renewed interest in the use of steroids in septic shock patients. Venkatesh et al. randomized 3658 patients with septic shock requiring mechanical ventilation to 200 mg/day continuous infusion of hydrocortisone or placebo for 7 days [7]. The study showed that hydrocortisone infusion did not result in a statistically significant reduction in 90-day mortality compared to placebo (27.9% vs. 28.8%). Patients in the hydrocortisone group had faster resolution of shock than those in the placebo (median duration of vasopressor use; 3 vs. 4 days, *p* < 0.001). Although patients in the hydrocortisone group had a shorter duration of the initial episode of mechanical ventilation, there was no significant difference in the number of days alive and free of mechanical ventilation). More adverse events were seen in the hydrocortisone group, even though the absolute incidence was low (1.1 vs. 0.3%, *p* = 0.009).

Another placebo-controlled trial was published in 2018 by Annane [8]. Similar to his first study, patients were randomized to receive a combination of hydrocortisone (50 mg IV every 6 h) with fludrocortisone (50 mcg once daily), administered for 7 days. A total of 1241 patients with septic shock for less than 24 h were enrolled. The primary outcome, death from any cause at day 90, was better in the treatment group (43.0 vs. 49.1%, relative risk 0.88, 95% CI 0.78 to 0.99, *p* = 0.03). Secondary outcomes also favored the use of steroids, including the 180-day mortality, vasopressor-free days, and organ failure-free days to day 28. The study showed more hyperglycemia in the steroids group, but similar rates of gastrointestinal bleeding and superinfections were seen.

Driven by these results, the 2024 SSC guidelines included a conditional recommendation in favor of low-dose corticosteroids in patients with ongoing shock following fluid resuscitation [9]. The debate regarding the association of low-dose corticosteroids with a mortality benefit in patients with septic shock and whether adding fludrocortisone has any additional benefit has yet to be resolved.

**Table 1:** Corticosteroids in Septic Shock.

Author or Trial Name (Year)	Population	Steroid Regimen Studied	Results
Luce (1988) [4]	− 75 patients within 1–2 h of admission with or development of shock with positive blood culture	− Methylprednisolone 30 mg/kg for four doses	− No difference in mortality or development of ARDS
Annane (2002) [5]	− 300 patients within 8 h of meeting criteria for septic shock	− Hydrocortisone 50 mg every 6 h and Fludrocortisone 50 mcg daily	− Improved 28-day mortality, primarily in patients without response to an ACTH stimulation test
Sprung (2008) [6]	− 499 patients up to 72 h after development of shock	− Hydrocortisone 50 mg every 6 h	− No difference in survival − Improved time to resolution of shock in patients who recovered − Increased adverse events
Venkatesh (2018) [7]	− 3658 patients with septic shock requiring mechanical ventilation for 4 to 24 h	− Hydrocortisone 200 mg/day as an infusion for 7 days	− No difference in survival − Improved time to shock resolution in the treatment group
Annane (2018) [8]	− 1241 patients with septic shock for under 24 h	− Hydrocortisone 50 mg every 6 h and Fludrocortisone 50 mcg daily	− Improved 90-day mortality, 180-day mortality, vasopressor-free days, and organ failure-free days to day 28 − Increased hyperglycemia in the treatment group.

## 3. Steroids in Acute Respiratory Distress Syndrome

ARDS is an intense inflammatory process that occurs at the level of the alveoli, alveolar blood membrane, and pulmonary interstitium. The alveolar space fills with fluids, cellular debris, and inflammatory cytokines [10]. This results in severe gas exchange abnormalities with subsequent development of hypoxia, with hypercapnia in severe cases. The inflammation in ARDS is dynamic. The initial stage of the disease, usually occurring during the first week, is characterized by the development of edema and hyaline membranes. The second phase, beginning in the second week, is characterized by the presence of interstitial inflammation and the development of interstitial fibrosis [11].

The use of steroids in ARDS has been investigated for decades (Table 2), and was proposed as a potential treatment in the original description of ARDS in 1967 [12]. One of the first randomized trials to use steroids in the treatment of ARDS was published by Bernard in 1987. They investigated the use of high-dose (30 mg/kg every six hours) methylprednisolone for 24 h in 99 patients with early ARDS [13]. Patients were enrolled early, within 7 to 11 h after meeting ARDS criteria. The patients were on mechanical ventilation for an average of 2 to 3 days prior to entry into the study. No difference in survival at 45 days was seen between the two groups. The rate of hypoglycemia was increased in the intervention group, but the infectious complications were similar. Importantly, this trial was conducted prior to the widespread use of lung-protective ventilation, which has become the cornerstone of ARDS management. Studies conducted since widespread adoption of lung-protective ventilation strategies have been inconsistent.

A decade later, Meduri evaluated the use of a more prolonged course of methylprednisolone, given to patients with ARDS at a later stage of the disease [14]. They enrolled patients who failed to improve after 7 days of respiratory failure. A total of 24 patients were randomized in a 2:1 ratio to methylprednisolone (loading dose of 2 mg/kg, followed by 2 mg/kg/day then a tapering regimen) or placebo. The treatment was continued for up to 32 days. Bronchioloalveolar lavage (BAL) was carried out on day 5 in order to detect the development of pneumonia. The study’s primary endpoint was the change in the Lung Injury Score (LIS), a combination of criteria including radiographic, gas exchange, positive end expiratory pressure (PEEP), and respiratory system mechanics [15]. Secondary endpoints included mortality and the multiorgan dysfunction score. The study found a significant improvement in the LIS favoring the intervention group. Other findings included improvement in the ICU mortality as well as the in-hospital mortality. The infection rate was similar in the two groups.

In 2006, and as a result of the encouraging preliminary data, the ARDS network conducted a trial in late ARDS, defined as 7 to 28 days after the onset of disease [16]. The study included patients with moderate-to-severe disease, defined as a PaO_2_/FiO_2_ < 200 mm Hg. Methylprednisolone was started with a loading dose of 2 mg/kg of predicted body weight and was then tapered over several weeks. Similar to the Meduri study, BAL was performed at 7 days to evaluate for pneumonia. The primary endpoint was the 60-day mortality, which was found to be similar between the two groups (29.2 vs. 28.6%, *p* = 1.0). However, a subgroup analysis of patients enrolled beyond 14 days of ARDS onset found an increased risk of death with methylprednisolone (35 vs. 8%, *p* = 0.02). The number of ventilator-free days at 28 days favored the intervention group (11.2 ± 9.4 vs. 6.8 ± 8.5, *p* < 0.001). The fact that the improvement in ventilator-free days did not translate into improvement in mortality was thought to be due to a high rate of reintubation, although it is worth noting that the PaO_2_/FiO_2_ and plateau pressure both improved in the treatment group. In terms of potential complications, the rate of suspected or probable pneumonia and shock were found to be lower in the intervention group. However, the intervention group had an increased risk of myopathy or neuropathy compared to the placebo group.

A year later, in a placebo-controlled trial enrolling 91 patients, Meduri re-assessed the use of steroids in early severe ARDS, using an infusion of methylprednisolone started within 72 h of disease onset. Starting at a lower dose of 1 mg/kg/day, the infusion was continued for up to 28 days [17]. Serial BALs were performed to monitor for infections. The primary endpoint, the LIS at day 7, improved more rapidly in the intervention group. A similar pattern of improvement was seen with the C-reactive protein (CRP). The duration of mechanical ventilation was significantly lower in the intervention group (5 vs. 9.5 days), as well as the length of ICU stay (7 vs. 14.5 days). A trend toward improved hospital mortality was seen in the steroid group. However, this was potentially confounded by a higher number of patients with shock included in the placebo group (46% vs. 24%). Fewer infections were seen in the treatment group, but an equal number of patients developed neuromuscular weakness. Of note, the study’s protocol called for avoidance of neuromuscular blocking agents, which may explain the differences compared to the ARDS network trial in terms of development of neuromuscular weakness.

A 2020 open-label randomized trial by Villar evaluated the use of dexamethasone in 277 patients with early ARDS [18]. Patients with early ARDS, defined as less than 30 h from onset, were enrolled. Dexamethasone was started at 20 mg/day for 5 days, then tapered to 10 mg/day for up to a total of 10 days. The study was stopped early due to low enrollment. The primary outcome was the number of ventilator-free days at 28 days, which was significantly better in the dexamethasone group (12.3 vs. 7.5 days). Additionally, all-cause mortality at 60 days, ICU mortality, and hospital mortality all favored the intervention group. The rate of adverse events was equal between the two groups.

In 2021, Lin published a meta-analysis assessing the use of steroids in ARDS patients. The analysis included a study with COVID-19 patients [19]. The mortality, ventilator-free days at day 28, and PaO_2_/FiO_2_ were all found to be improved in patients who received steroids [20]. A trend toward more hyperglycemia was seen in the steroid group, but the treatment group was again found to have a lower rate of infection.

The Society of Critical Care Medicine and the European Society of Intensive Care Medicine’s guidelines suggest the use of corticosteroids in patients with moderate-to-severe ARDS (PaO_2_/FiO_2_ < 200) within 14 days of disease onset [21]. However, this recommendation was conditional and was based on moderate quality of evidence. The same guidelines recommended surveillance to ensure identification of any hospital-acquired infection during therapy.

It is important to note that ARDS is a heterogenous process and likely represents a common endpoint for many inflammatory diseases. As such, a variety of diseases present with ARDS, or mimic ARDS, and are known to respond to steroids. These include but are not limited to: COVID pneumonia (discussed further in Section 5), vasculitis with diffuse alveolar hemorrhage, acute eosinophilic pneumonia, cryptogenic organizing pneumonia, nonspecific interstitial pneumonitis, acute hypersensitivity pneumonitis, pneumocystis jiroveci pneumonia, and pneumonitis associated with connective tissue disease. Treatment of these processes with steroids is strongly recommended [22]. It is also possible that there is a role for glucocorticoids in other specific conditions [23].

**Table 2:** Corticosteroids in ARDS.

Author or Trial Name (Year)	Population	Steroid Regimen Studied	Results
Bernard (1987) [13]	− 99 patients within 7 to 11 h after meeting ARDS criteria − Patients were on mechanical ventilation for an average of 2–3 days prior to entry into the study	− Methylprednisolone 30 mg/kg every 6 h for 24 h	− No difference in mortality at 45 days − Increased rate of hyperglycemia − No difference in infections
Meduri (1998) [14]	− 24 patients with ARDS who failed to improve after 7 days of respiratory failure	− Methylprednisolone loading dose of 2 mg/kg, followed by 2 mg/kg/day then a tapering regimen continued up to 32 days	− Improved Lung Injury Severity score (LIS) − Improved ICU and in-hospital mortality
Steinberg (2006) [16]	− 180 patients with moderate-to-severe disease (PaO_2_/FiO_2_ < 200) − Patients were enrolled 7–28 days after onset of ARDS	− Methylprednisolone 2 mg/kg of predicted body weight for 14 days then tapered slowly	− No difference in mortality overall, but increased mortality in patients enrolled beyond 14 days from disease onset
Meduri (2007) [17]	− 91 patients within 72 h of ARDS diagnosis	− Methylprednisolone 1 mg/kg for up to 28 days	− Improvement in the LIS, duration of mechanical ventilation, and ICU length of stay
Villar (2020) [18]	− 277 patients within 30 h of ARDS diagnosis	− Dexamethasone 20 mg/day for 5 days, then 10 mg/day for up to 10 days	− Stopped early due to low enrollment − Improved ventilator-free days at 28 days
Lin (2021) [20]	− Meta-analysis of nine studies with 1371 patients, many of whom had COVID-19	− Varied	− Improved mortality, ventilator-free days at 28 days, and PaO_2_/FiO_2_ ratio

## 4. Steroids in Severe Pneumonia

As with the other conditions covered in this review, severe community-acquired pneumonia (CAP) is characterized by inflammation due to increased pulmonary and circulating inflammatory cytokines. For example, a persistent elevation of interluken-6 has been seen in studies of patient who do not survive the infection compared to patients who do [24]. This again provides justification for the use of steroids in treatment of this disease. Table 3 includes 5 randomized trials that addressed the potential use of steroids in severe pneumonia.

In 2005, Confalonieri published a pilot placebo-controlled study of 46 ICU patients with severe CAP [24]. The severity was based on oxygenation, hemodynamic, and radiographic criteria. Hydrocortisone was started with a 200 mg bolus, followed by an infusion at 10 mg/h for a total of 7 days. At baseline, most patients were on mechanical ventilation, but few were in septic shock. Compared to placebo, the hydrocortisone group had a significant improvement in the PaO_2_/FiO_2_ and in the multiorgan dysfunction score at day 8. The CRP was also significantly lower in the steroid group, and the ICU and in-hospital mortality rates were lower in patients treated with hydrocortisone. More adverse events were seen in the placebo group, which suggested that hydrocortisone was safe in these patients.

In 2011, another small study assessed the effect of methylprednisolone in a population of patients with CAP who were not intubated [25]. In this study, 56 patients with respiratory failure (defined as a PaO_2_/FiO_2_ < 300) were enrolled. Methylprednisolone was given as a 200 mg bolus prior to the start of antibiotics and was followed by a tapering regimen for 9 days. The need for mechanical ventilation was the primary outcome. There was no difference between the steroid and placebo groups, but the number of events was small (1 and 5 cases, respectively). Initially, the PaO_2_/FiO_2_ favored the steroid group, but by day 7, there was virtually no difference between the groups. The decrease in IL-6 and CRP was found to be significantly quicker in the steroid group, and again there were few complications found to be related to their use.

A larger placebo-controlled trial was published in 2015 by Torres et al., evaluating the use of methylprednisolone in severe CAP [26]. Patients were eligible if they had a CRP greater than 150 mg/L, a level thought to increase the chance of recruiting patients with high inflammatory response. One hundred and twenty patients were enrolled and received methylprednisolone (0.5 mg/kg every 12 h) or placebo for 5 days, started within 36 h of hospital admission. The primary endpoint was defined as treatment failure, a combination of clinical and radiographic criteria. Most patients were admitted to the ICU, but few were on mechanical ventilation. The proportion of patients with septic shock was lower in the steroid group. There was no difference in the rate of early treatment failure (occurring up to 72 h), but the rate of late treatment failure was significantly lower in the methylprednisolone group (3% vs. 25%). However, the difference was primarily driven by a better radiographic progression in the steroid group, and the in-hospital mortality was not different between the groups. This study also identified a more rapid decrease in the level of CRP and IL-10. No association with superinfections or other adverse events was identified in the patients treated with steroids.

A VA cooperative study published in 2022 assessed the use of a lower-dose methylprednisolone in critically ill patients with severe CAP and healthcare-associated pneumonia (HCAP) [27]. This multicenter placebo-controlled trial enrolled 584 ICU and stepdown patients, within 72 to 96 h of hospital admission. Following a loading dose of 40 mg, methylprednisolone was continued for 20 days. The trial was stopped early due to low recruitment. Thirty-four percent of the patients had HCAP, and thirty-three percent were on mechanical ventilation. The study found no difference in the probability of survival at 60 days. Subgroup analysis found no difference in patients with HCAP. In addition, there was also no difference in the development of shock, ARDS, or ICU stay.

In early 2023, Dequin published the largest study to date assessing the use of hydrocortisone in 800 patients with severe CAP [28]. ICU patients in need of mechanical ventilation (invasive or noninvasive), a need for oxygen with a PaO_2_/FiO_2_ < 300, or a pulmonary severity index greater than 130 were enrolled. Hydrocortisone at 200 mg daily for 4 days was used, tapered for a total of 8 or 14 days based on clinical improvement. The steroids were administered early, with an average time between admission to the ICU and the first administration of less than 15 h. Influenza pneumonia and septic shock patients were excluded. The study was stopped after the second interim analysis. The most common organism isolated in this study was Streptococcus pneumonia. The primary outcome, 28-day mortality, was significantly lower in the hydrocortisone group (6.2 vs. 11.9). Secondary outcomes, including death by day 90, the cumulative incidence of endotracheal intubation in patients not receiving it at baseline, and the cumulative incidence of initiation of vasopressor in patients not on vasopressor at baseline, were also lower in the hydrocortisone group. Steroid use was not associated with an increase in hospital-acquired infections or GI bleeding, but a higher dose of insulin during the first 7 days of treatment was required in the patients treated with steroids.

Thus, the evidence supporting the use of steroids in severe community-acquired pneumonia appears relatively strong when hydrocortisone infusion is administered early. The benefits of methylprednisolone are less clear. However, both agents appear to have a good safety profile in this population. It is worth noting that the data on nosocomial pneumonia are very limited, and the safety of steroids in influenza pneumonia remains in question [29].

## 5. Steroids in COVID-19

Glucocorticoids were among the first therapies studied in the management of patients with severe SARS-CoV-2 infection (COVID-19). Inflammatory markers and cytokines including CRP, ferritin, IL 1, and IL 6 are elevated in COVID-19 and correlate with the intensity of the disease. While the role of steroids in ARDS at the onset of the COVID-19 pandemic was in question, severe COVID-19 was noted to have distinct radiographic features compared to ARDS from other etiologies. Diffuse ground glass opacities constitute the predominant feature in COVID-19 pneumonia [30], compared with basilar and lower lobe consolidations, and relatively normal and unaffected anterior and apical areas, which is more traditionally seen in ARDS [31].

Three randomized controlled trials evaluated the use of corticosteroids in COVID-19 (Table 4). The largest and earliest study was the RECOVERY trial [32]. This randomized open-label study evaluated the use of oral or IV dexamethasone at a dose of 6 mg once daily for up to 10 days. It included 6425 patients hospitalized with COVID-19. The primary outcome was the 28-day mortality. Overall, a significant improvement was seen in this endpoint (rate ratio 0.83). However, the effect depended on the severity of the disease. In patients who did not need oxygen therapy at baseline, no improvement was seen with the use of dexamethasone. However, patients who were on oxygen without invasive ventilation saw a significant improvement in mortality with the use of steroids (23.3% vs. 26.2%; rate ratio 0.82). Patients receiving invasive mechanical ventilation saw the most significant benefit from the use of dexamethasone (29.3% vs. 41.4%; rate ratio 0.64). This translated into an absolute reduction in 28-day mortality of 12.3% in patients on invasive mechanical ventilation and 4.2% in those receiving oxygen therapy without invasive ventilation. Therapy with glucocorticoids was safe overall, with only 4 serious adverse reactions deemed related to dexamethasone (2 hyperglycemias, 1 gastrointestinal hemorrhage, and 1 psychosis). Based on this landmark trial, dexamethasone quickly became a standard of care in patients hospitalized with hypoxic respiratory failure due to COVID-19 pneumonia.

Other randomized trials evaluating the use of steroids in COVID-19 were stopped early based on ethical grounds once the RECOVERY trial was published. The first was a placebo-controlled trial conducted in France by Dequin et al., evaluating the use of hydrocortisone infusion (200 mg/day) in patients with COVID-19 requiring ICU admission, who met prespecified severity criteria [33]. A trend toward lower 21-day mortality in the steroid group was seen (14.7 vs. 27.4%). No difference in the incidence of nosocomial pneumonia was seen in this trial.

The second was the CODEX randomized trial, conducted in 41 ICUs in Brazil. In this open-label study, Tomazini et al. evaluated a higher dose of dexamethasone (20 mg of IV dexamethasone daily for 5 days, then 10 mg daily for 5 days or until ICU discharge) in patients receiving mechanical ventilation for COVID-19-related ARDS. Patients were enrolled in the first 48 h of meeting moderate-to-severe ARDS criteria (PaO_2_/FiO_2_ < 200) [19]. This study was also terminated early based on ethical grounds. The 28-day mortality was not different between the two groups, However, the number of ventilator-free days at 28 days favored the steroid group (6.6 vs. 4.0 days). The incidence of adverse events was again similar between the two groups.

Finally, a 2020 meta-analysis evaluating the association between systemic steroids and mortality among critically ill patients with COVID-19 found a significant difference in favor of the use of steroids, with no difference in the risk of serious or adverse events [34].

As such, steroids are now considered a mainstay of therapy in patients with severe COVID-19 who require oxygen or ventilatory support.

## 6. Steroids in Hypercapnic Respiratory Failure Due to Chronic Obstructive Pulmonary Disease

Systemic steroids are used extensively in the treatment of hypercapnic respiratory failure due to COPD. Their use is common based on the idea that airway inflammation underlies the acute exacerbation and results in air trapping that impairs ventilation. Despite the widespread use of steroids in hypercapnic respiratory failure from COPD, there is surprisingly little evidence supporting it. One of the first randomized trials evaluating this population came from a 1999 Veterans Affairs cooperative study [35]. In this trial, methylprednisolone (for 2 or 8 weeks) was compared to placebo in patients admitted for exacerbation of COPD. The study found a shorter initial hospitalization in the combined glucocorticoid group (9.7 vs. 8.5 days), in addition to a lower rate of first treatment failure at 30 days (23% vs. 33%) and 90 days (37% vs. 48%), but not 6 months. Few deaths and intubations were noted in the study. The rate of secondary infections was not different, but increased rates of hypoglycemia requiring treatment were seen in the steroid group.

To date, only two studies have assessed the use of steroids specifically in patients with hypercapnic respiratory failure from COPD (Table 5).

The first was a 2011 study by Alia, evaluating the effect of methylprednisolone when started within 24 h of ICU admission for acute hypercapnic respiratory failure [36]. This double-blind placebo-controlled trial enrolled 83 patients receiving ventilatory support (invasive or noninvasive) but was stopped early due to low recruitment. Methylprednisolone was started at 0.5 mg/kg every 6 h, and tapered over 10 days. Most patients were men, and exacerbations were mainly related to respiratory infections. Slightly less than half of the population was on noninvasive ventilation at baseline. This study found a significant reduction in the duration of mechanical ventilation in the corticosteroids group (3 vs. 4 days). The rate of noninvasive ventilation failure was significantly lower in the steroid group (0% vs. 37%). There was a trend toward a shorter median length of ICU stay, but no effect on mortality was found. In addition, the study was not able to find a difference in the rate of improvement of intrinsic PEEP (a measure of air trapping). The rate of PaCO_2_ decrease favored the steroid group only on days 2 and 3. More cases of hyperglycemia were noted in the steroid group, but there was no difference in other adverse events.

The second study was published by Abroug in 2014 [37]. This was an open-label randomized trial assessing oral prednisone (1 mg/kg daily for up to 10 days) in patients requiring invasive or noninvasive ventilatory support for acute hypercapnic respiratory failure. A total of 217 patients were enrolled in 2 Tunisian ICUs. The study was also stopped early due to low recruitment. Most patients were men and on home oxygen therapy. Seventy-six percent were on noninvasive ventilation at baseline. The ICU mortality was similar in the groups. Unlike the study by Alia, the rate of noninvasive failure did not differ between the groups. The duration of mechanical ventilation and ICU stay were also similar. More hyperglycemic events were seen with the steroid use.

Thus, while steroids are often thought of as a mainstay in the treatment of acute exacerbation of COPD with resulting hypercapnic respiratory failure, based on the limited data available, it is difficult to find strong evidence to support this practice. Ongoing studies will hopefully shed more light on this important and common clinical entity. It is important to note that this discussion of steroids in hypercapnic respiratory failure is limited to COPD, where the evidence is less certain. In patients with hypercapnic respiratory failure due to asthma, in particular asthma with a type 2 inflammatory phenotype, the role of steroids is clearer. However, even in the asthma population, management of patients requiring invasive or noninvasive ventilation has not been studied extensively, and much of the current practice is extrapolated from management in a broad emergency department population [38].

## 7. Conclusions

Glucocorticoids are widely used in the treatment of commonly encountered diseases in medical intensive care units. Strong data exist for the use of these medications in the management of certain specific etiologies, such as COVID-19. However, for broader problems that result as a common endpoint from many causes such as septic shock, ARDS, and CAP, the role of steroids is still being debated. The heterogenous nature of these diseases makes a uniform approach difficult. However, based on current data, it does appear that steroids do have a role in management. In hypercapnic respiratory failure from COPD, while steroids are commonly used, there are very few studies to support or refute this approach.

## Figures and Tables

**Table 3:** Corticosteroids in Severe Pneumonia.

Author or Trial Name (Year)	Population	Steroid Regimen Studied	Results
Confalonieri (2005) [24]	− 46 ICU patients with severe CAP	− Hydrocortisone 200 mg then 10 mg/h for 7 days	− Improved mortality, hospital length of stay, progression to septic shock, chest radiograph score, and PaO_2_/FiO_2_
Fernandez-Serrano (2011) [25]	− 56 patients with CAP not requiring intubation	− Methylprednisolone 200 mg bolus then tapered over 9 days	− No difference in need for mechanical ventilation
Torres (2015) [26]	− 120 patients with CAP and a CRP above 150 mg/L within 36 h of hospital admission	− Methylprednisolone 0.5 mg/kg every 12 h for 5 days	− No difference in hospital mortality − Improved radiographic progression in the treatment group
Meduri (2022) [27]	− 548 ICU and stepdown patients within 72 to 96 h of admission − Stopped early due to low recruitment	− Methylprednisolone 40 mg daily	− No difference in 60-day mortality
Dequin (2023) [28]	− 795 patients admitted to the ICU for CAP	− Hydrocortisone 200 mg daily for 4–7 days then tapered	− Improved 28-day mortality

**Table 4:** Corticosteroids in COVID-19.

Author or Trial Name (Year)	Population	Steroid Regimen Studied	Results
RECOVERY (2021) [32]	− 6425 with COIVID-19	− Dexamethasone 6 mg daily for up to 10 days	− Improved mortality (Effect was largest in patients on mechanical ventilation)
Dequin (2020) [33]	− 149 patients with COVID-19	− Hydrocortisone 200 mg daily	− Stopped early when results from RECOVERY were released
Tomazini (2020) [19]	− 299 patients with COVID-19 and moderate or severe ARDS	− Dexamethasone 20 mg daily for 5 days, then 10 mg for 5 days	− Stopped early when results from RECOVERY were released − Improved ventilator-free days

**Table 5:** Corticosteroids in COPD with hypercapnic respiratory failure.

Author or Trial Name (Year)	Population	Steroid Regimen Studied	Results
Alia (2011) [36]	− 83 patients with invasive or noninvasive ventilatory support − Stopped early due to low recruitment	− Methylprednisolone 0.5 mg/kg every 6 h then tapered over 10 days	− No difference in mortality − Significantly reduced duration of mechanical ventilation and failure of noninvasive mechanical ventilation
Abroug (2014) [37]	− 217 patients admitted to the ICU requiring ventilatory support	− Prednisone 1 mg/kg daily for up to 10 days	− No difference in mortality or mechanical ventilation − Increased hyperglycemic events
